# ‘*Is anyone else’s husband trying to undermine them all the time?*’: A reflexive thematic analysis of online support forum discussions about bariatric surgery saboteurs

**DOI:** 10.1177/13591053241305946

**Published:** 2024-12-29

**Authors:** Sophia Quirke-McFarlane, Jane Ogden

**Affiliations:** University of Surrey, UK

**Keywords:** bariatric surgery, online support forum, qualitative, sabotage, social support, thematic analysis

## Abstract

Online support forums (OSFs) are an increasingly utilised social support resource for bariatric surgery (BS) patients. OSFs could permit patients to discuss sensitive topics, such as being sabotaged post-BS. This study aimed to explore the phenomenon of BS saboteurs via BS-specific OSFs. Two internet search engines were used to identify BS-specific OSFs. The eligible OSF(s) was searched to identify relevant threads. Thread data were analysed using reflexive thematic analysis. One OSF was eligible. The final dataset included 18 threads (*N* replies = 569). Three themes were identified: *Feeder Behaviours*, *Negative Reactions to Bariatric Surgery-Induced Weight Loss* and *Strategies to Avoid and/or Manage Bariatric Surgery Saboteurs*. Transcending these was the notion of *The Online Support Forum as a Source of Substituted Social Support and Place of Solace*. In lieu of the limited social support received in real life, BS patients use OSFs as a form of social support when being sabotaged.

## Introduction

Bariatric surgery (BS) is considered the most effective weight loss intervention for people living with obesity (Colquitt et al., 2014; Picot et al., 2009; Torgerson and Sjöström, 2001). Patients typically lose between 42.6% and 61.5% of excess weight loss during the first year post-BS (Garb et al., 2009). Irrespective of these promising weight outcomes, many patients experience a range of psychological, physiological and social stressors and challenges in adapting to recommended BS behavioural changes (Lent et al., 2014; Lyons et al., 2014; Mechanick et al., 2013; Mitchell et al., 2012). Accordingly, research suggests that social support can help BS patients adapt to these changes (Ogle et al., 2016; Pories et al., 2016; Quirke-McFarlane and Ogden, 2024; Tolvanen et al., 2021).

Support groups are of particular importance and attendance at these groups has been shown to increase both short-term and long-term weight loss post-BS (Andreu et al., 2020; Beck et al., 2012; Livhits et al., 2011). Despite the advantages of participating in support groups, barriers such as mobility and transportation issues (Peytremann Bridevaux and Santos-Eggimann, 2008) and long travel distances (Kuo et al., 2015) can prevent BS patients from attending traditional in-person support groups. As a result, online support forums (OSFs) have become an increasingly utilised social support resource for BS patients (Athanasiadis et al., 2021; Atwood et al., 2018; Das and Faxvaag, 2014; Koball et al., 2017; Robinson et al., 2020; Willmer and Salzmann-Erikson, 2018).

OSFs are defined as online services with features that enable members to communicate with each other (Malinen, 2015). They provide a dynamic environment where individuals can discuss health-related topics, learn how others have managed problems and ultimately receive social support (Athanasiadis et al., 2021; Atwood et al., 2018; Das and Faxvaag, 2014; Koball et al., 2017; Robinson et al., 2020; Willmer and Salzmann-Erikson, 2018). Furthermore, OSFs are important mediums through which members can disclose personal information (Das and Faxvaag, 2014; Yang et al., 2017). Thus, OSFs could serve as a platform for patients to discuss sensitive topics such as being sabotaged post-BS.

The phenomenon of BS saboteurs was first studied by Andrews (1997). Through their case studies, Andrews (1997) described how partners sabotaged BS patients via ostracism, jealousy, paranoia, feeder behaviour, control, anger, as well as emotional and physical abuse. Ogden and Quirke-McFarlane (2023) have since furthered the understanding of this phenomenon through their new model of negative social support which consists of sabotage, feeder behaviour and collusion. Moreover, as evidenced by the wider literature (Azizi et al., 2020; Kershaw et al., 2014; Kouvonen et al., 2011; Quirke-McFarlane and Ogden, 2024), negative social support can undermine an individual’s weight loss and/or maintenance attempts. For instance, a recent qualitative study by Quirke-McFarlane and Ogden (2024) identified how some BS patients attributed their recurrent weight gain to their partner’s feeder behaviours.

To date, the existing BS OSF evidence base has explored topics such as the type, frequency and value of social support provided (Athanasiadis et al., 2021; Atwood et al., 2018; Das and Faxvaag, 2014; Koball et al., 2017; Robinson et al., 2020; Willmer and Salzmann-Erikson, 2018), with limited attention given to more sensitive topics, such as BS saboteurs. Therefore, the present study aimed to explore the phenomenon of BS saboteurs via BS OSFs, focusing specifically on the post-BS period. In this study, a BS saboteur is defined as an individual who undermines another person’s BS success, either unintentionally or intentionally. Specific research questions included:

1. What behaviours and/or comments, if any, do BS OSF members classify as forms of sabotage post-BS?
2. What types of social support, if any, are exchanged in response to BS OSF members’ experiences with BS saboteurs?

## Materials and methods

### Design

Given its ability to obtain insights into an individual’s experiences, behaviour, beliefs, attitudes and motivation (Oranga and Matere, 2023), a qualitative design was deemed apt.

### Data collection

#### Online support forum identification

Using relevant search terms (Supplemental Table 1), a comprehensive online search using two internet search engines was conducted to obtain a list of existing BS OSFs. Eligible OSFs were BS-specific, available in the English language, publicly available, active (defined by Willmer and Salzmann-Erikson (2018) as having 10 daily postings, >1000 postings total and >100 members), and had a search bar.

#### Thread identification

To identify relevant OSF threads, 50 search terms (Supplemental Table 2) related to sabotage were generated. First, all 50 search terms were searched on the previously identified OSF(s), noting the associated search results. Second, threads were organised from newest to oldest on a single thread page and the 10 search terms with the most thread search results were examined for eligibility. Search terms were deemed eligible if ≥5 threads on the thread page discussed behaviour(s) and/or comment(s) of a family member(s), friend(s) and/or acquaintance(s).

Following the identification of eligible search terms, the previously identified OSF(s) was searched without a specific posting period (Smedley and Coulson, 2021). Eligible threads were those where the original post in the thread related to received behaviours and/or comments perceived by the OSF member to undermine their post-BS success. A random sampling strategy was then employed to select threads for inclusion in the dataset, thereby ensuring each eligible thread had an equal probability of being selected (Smedley and Coulson, 2021). Eligible threads were assigned numbers, sorted numerically and randomly selected using a computer programme.

### Ethical considerations

This study was granted favourable ethical opinion by the University Ethics Committee (FHMS 21-22 059 EGA). The British Psychological Society’s (2021) ethics guidelines for internet-mediated research were also adhered to. As this study analysed publicly available threads from the eligible OSF(s), we did not consider it necessary to obtain informed consent from OSF members (Sudweeks and Rafaeli, 1996). Additionally, our analysis entailed observation and thus group processes were not affected (Winzelberg, 1997). Furthermore, to help prevent traceability and protect the privacy and anonymity/pseudonymity of OSF members, the name(s) of the eligible OSF(s) utilised was not disclosed and thread quotations were anonymised, paraphrased and checked using two internet search engines (Roberts, 2015).

### Data analysis

Secondary thread data were analysed using NVivo v12 (QSR International Pty Ltd). Given its flexible nature and ability to provide a rich and detailed, yet complex account of the data, an inductive approach to reflexive thematic analysis (Braun and Clarke, 2006, 2022) was employed. To identify themes within the thread data, Braun and Clarke’s (2022) six-phase reflexive thematic analysis framework was followed: familiarisation with the data; coding; generating initial themes; developing and reviewing themes; refining, defining and naming themes; and writing up. Data were analysed at both a semantic and latent level (Braun and Clarke, 2006). In line with Clarke and Braun’s (2013) recommendations, one researcher led the coding (SQM). However, ongoing iterative discussions were held within the research team throughout the data analysis process. A critical realist epistemological framework was adopted throughout data analysis (Braun and Clarke, 2022). Furthermore, the researcher held an insider-outside position, which afforded them a deeper knowledge, although not a complete understanding, of the experience being studied (Dwyer and Buckle, 2009).

## Results

### Online support forum identification

A total of 32 OSFs were identified from the comprehensive online search. Following the application of the eligibility criteria, only one OSF was deemed eligible. The ineligible OSFs were not BS-specific (*n* = 5), publicly available (*n* = 6), active (*n* = 8) or lacked a search bar (*n* = 12). At the time of data collection, the eligible OSF had >300,000 members, with more than 7 million monthly visits. Members were required to create an account to use and post on the eligible OSF. To the researcher’s knowledge, no other checks to remove bots were taken.

### Thread identification

**Figure 1.**
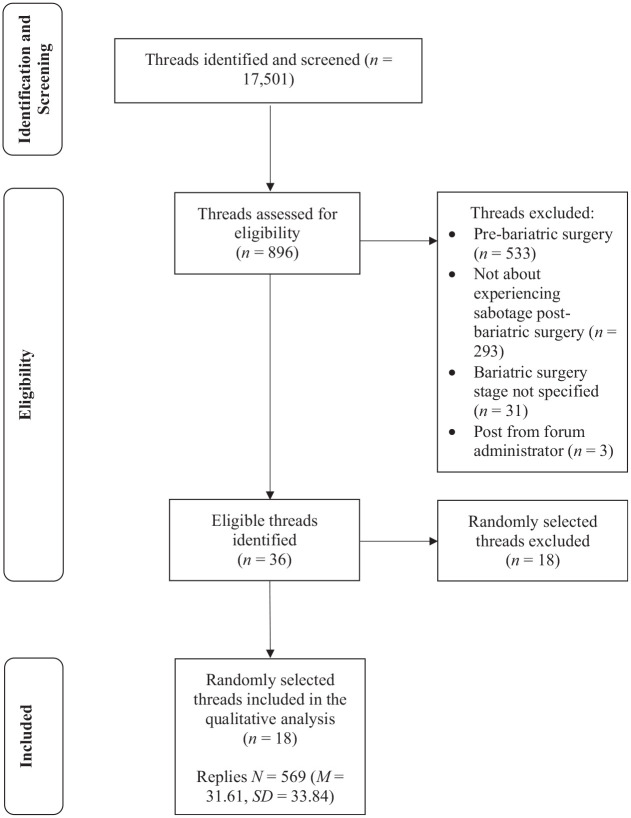
Flowchart detailing the selection of threads.

In addition to sabotage and saboteur, following the application of the thread search term eligibility criteria, a further four search terms (insecure, negative, jealous and hurtful) were deemed eligible to search the OSF. Together, the six search terms resulted in the generation of 17,501 threads (Figure 1). Following the screening of thread titles and associated excerpts, 896 threads were classified as potentially eligible. After an in-depth examination, 860 of the 896 threads were excluded (Figure 1 lists the reasons), leaving 36 eligible threads for potential inclusion in the dataset. The 36 eligible threads identified had 1026 replies, ranging from 4 to 146 replies per thread (*M* = 28.50, SD = 33.13). Due to the vast amount of data identified, 50% of threads were randomly selected for inclusion, thereby leaving 18 threads for inclusion in the final dataset. The 18 threads included in the final dataset had 569 replies, ranging from 9 to 144 replies per thread (*M* = 31.61, SD = 33.84).

### Overview of themes identified

**Figure 2.**
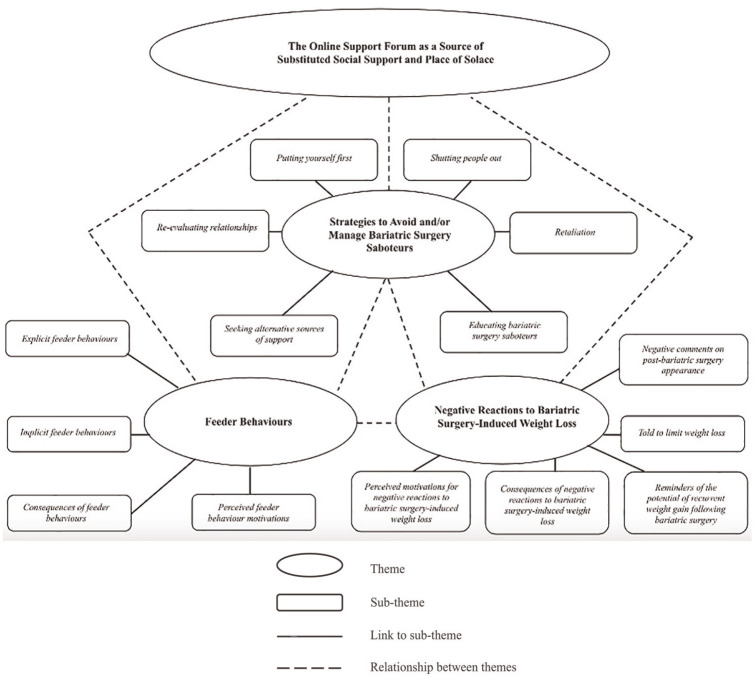
Thematic map of the themes and sub-themes identified from the reflexive thematic analysis of the data.

Three main themes were identified from the data: *Feeder Behaviours*, *Negative Reactions to Bariatric Surgery-Induced Weight Loss* and *Strategies to Avoid and/or Manage Bariatric Surgery Saboteurs*. Transcending these was the theme *The Online Support Forum as a Source of Substituted Social Support and Place of Solace* (Figure 2).

### Theme 1: Feeder Behaviours

Many OSF members disclosed incidents of sabotage via *Feeder Behaviours*, with some OSF members describing this behaviour as a common phenomenon post-BS:

You’re describing a phenomenon that can take many different forms and that is not uncommon here [on the bariatric surgery online support forum].

A variety of ‘*feeders*’ were reported, ranging from family and friends to acquaintances (e.g. work colleagues). OSF members described such *Feeder Behaviours* as being explicit or implicit and provided a variety of consequences of and potential explanations for these behaviours.

#### Explicit feeder behaviours

OSF members suggested that some *Feeder Behaviours* were explicit (i.e. physically and/or verbally articulated). The most common explicit feeder behaviour described by OSF members included physically being offered foods deemed unhealthy:

Is anyone else’s husband trying to undermine them all the time? […] My husband frequently brings snacks, such as chips and cookies, to bed and offers them to me […] It’s only been a couple of weeks since I’ve had bariatric surgery!

An additional form of explicit feeder behaviour revealed by OSF members included their family and friends’ tendency to verbally persuade them via enabling comments to consume foods deemed unhealthy:

Every time I visit my mom, she still offers me junk food and tells me ‘Oh, one bite won’t hurt you!’. Sure, you’ve been saying that for the last 34 years…

#### Implicit feeder behaviours

OSF members also described *Feeder Behaviours* of a more implicit nature (i.e. implied behaviours). Examples included partner’s tendency to bring foods deemed unhealthy into the home environment:

Well, pre- and post-bariatric surgery, my ex-partner never supported my lifestyle change. He continued to bring unhealthy foods, such as fast food and sweets, into the house.

Another instance of an implicit feeder behaviour cited by several OSF members involved partners consuming foods deemed unhealthy in front of them:

Since I’ve had my bariatric surgery, my wife has gone to the McDonald’s drive-thru three times. When we have movie nights, she breaks out bags of chips and other unhealthy snacks. It seems as if she has little regard for my new diet and lifestyle.

#### Consequences of feeder behaviours

OSF members reported several psychological and physiological consequences of *Feeder Behaviours*. In addition to negative feelings of ‘*stress*’ and ‘*betrayal*’, challenges to one’s willpower was a further psychological consequence cited by some OSF members:

While we were running errands, my husband stopped at McDonald’s and got a full meal because he was hungry […] For a taste of that burger, I would have sold my soul, killed my mother, and given up my firstborn.

While some OSF members were able to maintain their willpower around food triggers, others, conversely, succumbed and experienced physiological consequences, such as recurrent weight gain:

As a result of my mom’s feeder behaviour, I experienced significant weight regain after my [bariatric] surgery.

#### Perceived feeder behaviour motivations

Many OSF members also discussed perceived motivations for their family, friends and acquaintances’ *Feeder Behaviours*. While some OSF members found it difficult to comprehend the perceived motivations behind *Feeder Behaviours*, others, in contrast, were able to articulate a variety of perceived motivations. Perceived unintentional motivations for *Feeder Behaviours* included affection, guilt avoidance, lack of education about BS, nutritional concern and routine. Of these, affection was the most commonly cited perceived unintentional motivation underpinning *Feeder Behaviours*:

He might also be attempting to show you how much he loves and cares for you by gifting you treats and snacks.

However, some OSF members questioned whether any motives behind *Feeder Behaviours*, particularly those underpinned by affection, are truly innocent. They suggested that such actions might sometimes be a subtle form of undermining someone’s dietary adherence:

It’s sweet of you to attempt to give her husband the benefit of the doubt, but to me, it seems like textbook sabotage. He cannot possibly be unaware that certain foods are off-limits to her at this point.

In contrast, other perceived motivations for *Feeder Behaviours* were classified as intentional. These included envy, insecurity, jealousy, own weight management, preference for women with higher weight and spite. Envy, insecurity and jealousy were frequently cited as potential motivations behind *Feeder Behaviours*, thereby suggesting that some individuals may intentionally undermine a loved one’s dietary efforts due to negative emotions:

I am aware that most of the time it [feeder behaviour] stems from jealousy as you had the guts to confront the situation head-on and make healthier lifestyle choices.

This theme described sabotage in terms of *Feeder Behaviours.* OSF members described such *Feeder Behaviours* as being explicit or implicit and provided a variety of consequences of and perceived motivations for *Feeder Behaviours*.

### Theme 2: Negative Reactions to Bariatric Surgery-Induced Weight Loss

An additional form of sabotage described by OSF members was *Negative Reactions to Bariatric Surgery-Induced Weight Loss*. Although these negative reactions were predominately from family and friends, in some cases they also came from acquaintances. Negative reactions included negative comments on their post-BS appearance, being told to limit weight loss, as well as reminders about the potential of recurrent weight gain following BS. Furthermore, OSF members provided a range of consequences of and potential motivations for *Negative Reactions to Bariatric Surgery-Induced Weight Loss*.

#### Negative comments on post-bariatric surgery appearance

Many OSF members revealed that some of their family, friends and acquaintances made negative comments about their post-BS appearance, including being told they now looked ‘*sickly*’, ‘*aged*’, like a ‘*crackhead*’ or that they have ‘*cancer*’:

My friend told me I looked ‘sickly’, that ‘50 lbs is plenty’, and when I end up where I started in a few years, I would regret ‘butchering my body’.

Further, a few OSF members recounted being made aware, either explicitly or implicitly, by some of their family and friends that they preferred the pre-BS version of them:

My friend who arrived first screamed in shock, ‘Oh my god! Oh my god! What happened?!’, to which I responded, ‘I had bariatric surgery’. Following this, she paused and said, ‘I want the old you back’.

#### Told to limit weight loss

Given the negative comments on their post-BS appearance, some OSF members admitted that family and friends told them to limit their weight loss:

My husband genuinely told me that I should stop losing weight since I am beginning to resemble a ‘crackhead’.

Comments like this were not only common after significant BS-induced weight loss, with one OSF member disclosing that their partner made similar remarks 5 days post-BS:

I’m exactly five days post-bariatric surgery. I’m a hot mess: I have stitches, I’m on painkillers, and I’m learning to eat. My sweet hubby then revealed, ‘You know, I’m not really drawn to skinny women. I really hope you don’t lose a lot of weight’. Wtf?!.

#### Reminders about the potential of recurrent weight gain following bariatric surgery

Several OSF members recounted how family, friends and acquaintances frequently reminded them, be it explicitly or implicitly, about the potential of recurrent weight gain following BS. Explicit reminders included references to people who experienced recurrent weight gain following BS:

My sister and a close friend, who are both living with obesity, reacted really negatively to my decision to get bariatric surgery. As a result, both relationships have suffered. Both referenced people who underwent bariatric surgery and experienced weight regain.

Implicit reminders of the potential of recurrent weight gain following BS included indirect references to their past weight loss/maintenance failures:

When my parents informed my sister that I had shed 50 lbs in answer to her text about how I was doing, she replied ‘Let’s see how long that lasts’ … .

#### Consequences of negative reactions to bariatric surgery-induced weight loss

OSF members reported several consequences of *Negative Reactions to Bariatric Surgery-Induced Weight Loss*. For instance, many OSF members reported psychological consequences, including reduced motivation and self-esteem, as well as feelings of depression and hopelessness:

My partner continues warning me not to lose much else weight since I already look awful […] Hearing him say these things can be depressing for me because I have been overweight my entire life. Why I choose to see the good in this process is beyond me…

For some OSF members, these psychological consequences had subsequent psychological and physiological consequences, such as emotional eating:

I’m just sick and tired of being put down, and of course, when I’m stressed or upset, I turn to food to cope.

Moreover, several OSF members revealed experiencing strained relationships with their family and friends due to their negative reactions to their BS-induced weight loss. For example, many OSF members reported feeling ostracised as a result of their weight loss from BS:

My best friends were incredibly supportive up until they saw me in person. Since then, they’ve dropped like flies.

In discussions about romantic relationships, OSF members widely recognised that BS and its associated changes can lead to relationship breakdowns. Many expressed concerns about the potential for separation or divorce, highlighting the complex emotional landscape that accompanies such significant physical transformations:

The truth is that many relationships end after the other partner has had bariatric surgery. Search this forum if you don’t trust me. There are numerous instances of couples who split up or got divorced as a direct result of the surgery’s success. Often, it’s not because of the surgery itself, but because of what it represented.

#### Perceived motivations for negative reactions to bariatric surgery-induced weight loss

OSF members provided a variety of perceived motivations for negative reactions to their BS-induced weight loss. Being caught off guard and equating weight loss to a change in personality were the only perceived motivations underpinning *Negative Reactions to Bariatric Surgery-Induced Weight Loss* that OSF members classified as innocuous:

It appears that your friend was merely surprised that you had lost a significant amount of weight and may have not meant any harm by her reaction […] Because some people associate substantial weight loss with a personality shift, she might have believed you changed.

Conversely, there was widespread agreement that the other perceived motivations for *Negative Reactions to Bariatric Surgery-Induced Weight Loss* reported were of malicious intent, one of which was difficulties with adjusting to changes in the status quo:

People in our lives frequently have no interest in seeing us thrive because it changes the dynamics of the relationship.

For partners, insecurity often manifested in distinct ways, fuelled by the looming threat of a potential breakdown in the relationship. OSF members implied that these insecurities stemmed from fears that significant weight loss could alter the relationship dynamics, resulting in feelings of inadequacy or abandonment:

I hate to say it, but his insecurity is the driver behind his behaviour. He probably fears that if you lose a significant amount of weight, you will not want him around anymore. He is trying to project his own insecurities onto you in order to prevent you from succeeding.

Similarly, some OSF members noted that both jealousy and a perceived loss of control significantly influenced partners’ negative reactions to their weight loss following BS. OSF members suggested that these feelings stemmed from a partner’s struggle to accept the shift in dynamics:

He is jealous because he is no longer your enabler! You now possess the power and are in control of your success.

From this theme, it is evident that sabotage also took the form of *Negative Reactions to Bariatric Surgery-Induced Weight Loss*. Negative reactions included comments on their post-BS appearance, being told to limit weight loss, and reminders about the potential of recurrent weight gain. Additionally, OSF members provided a range of consequences of and perceived motivations for these reactions.

### Theme 3: Strategies to Avoid and/or Manage Bariatric Surgery Saboteurs

Multiple *Strategies to Avoid and/or Manage Bariatric Surgery Saboteurs* were provided by OSF members. These included shutting people out, retaliation, educating BS saboteurs, seeking alternative sources of support, re-evaluating relationships and putting yourself first.

#### Shutting people out

To prevent being sabotaged via *Feeder Behaviours* and *Negative Reactions to Bariatric Surgery-Induced Weight Loss*, several OSF members reported shutting people out. One way in which this was done was by only informing trusted others about their BS:

This is the main reason I keep my bariatric surgery a secret from people – it’s none of their concern, and I don’t need any negative attention.

Another way in which OSF members shut people out was by ignoring BS saboteurs sabotaging attempts. While this coping strategy was provided in response to *Feeder Behaviours*:

Your aunt will stop [sabotaging] if you ignore the food pictures she sends you.

It was more commonly offered in response to *Negative Reactions to Bariatric Surgery-Induced Weight Loss*:

My advice is to be like me, an ice queen, and ignore them [saboteurs] because much of what they have to say is pointless. After all, we all did this for ourselves in order to improve our health, not for any other purpose.

#### Retaliation

Retaliation was another strategy for managing BS saboteurs proposed by a few OSF members. For *Feeder Behaviours*, the retaliation strategies suggested were both verbal and physical in nature:

If you’re still with him on his birthday, buy yourself something, and when he asks why you gave him something he can’t use, just reply ‘Sort of like the chocolates for me’. Or you could keep the chocolates and regift them to him next Valentine’s Day.

On the contrary, for *Negative Reactions to Bariatric Surgery-Induced Weight Loss*, the retaliation strategies offered were verbal and often less extreme:

Call him out on it next time he says something like that. It doesn’t need to be accusatory or confrontational, just a calm even-tempered question. Is it satisfying for him to hurt your feelings? Why does he care more about your looks than your health?

#### Educating bariatric surgery saboteurs

To manage BS saboteurs more effectively, several OSF members emphasised the importance of educating family, friends and acquaintances. In response to *Negative Reactions to Bariatric Surgery-Induced Weight Loss*, a few OSF members advised others to provide their family and friends with reassurance:

Reassure your mom that you are trying to live a healthy lifestyle and that your weight loss efforts are for a better you.

Regarding both *Feeder Behaviours* and *Negative Reactions to Bariatric Surgery-Induced Weight Loss*, some OSF members recommended educating family and friends about BS by themselves or via support groups:

If they [support network] are simply ignorant, try explaining to them why you have to make different food choices, the size of your stomach, and other relevant bariatric surgery information.

Regarding *Feeder Behaviours*, to prevent receiving foods deemed unhealthy in the future, a few OSF members advocated viewing such situations as teachable moments:

Next time your boyfriend stops for a milkshake, ask if he can fetch you a Greek yoghurt or even a vitamin water. Simply request a healthier substitute.

Alternatively, creating a list of BS-friendly foods was another strategy on how to prevent receiving food gifts deemed unhealthy in the future and ultimately educate family and friends:

For food-centric holidays, like Valentine’s Day and Easter (if celebrated), I would make your boyfriend a list of really beloved food products, such as your favourite coffee syrup, protein powder, or water enhancer.

#### Seeking alternative sources of support

Seeking alternative sources of support was a further strategy mentioned to mitigate and/or manage BS saboteurs. Regarding *Feeder Behaviours*, some OSF members suggested expanding their support network:

By no means have I abandoned my former pals, but I have also made some new friends.

Regarding *Negative Reactions to Bariatric Surgery-Induced Weight Loss*, an alternative source of support suggested was attending counselling. Some OSF members indicated that this approach offered a supportive environment for navigating complex emotions post-BS:

One thing I would advise is that you get counselling to help you deal with your feelings and, ideally, gain advice on how to handle unsupportive friends and relatives.

For *Negative Reactions to Bariatric Surgery-Induced Weight Loss* motivated by partner insecurity, several OSF members recommended seeking couples counselling:

Would your husband be open to attending a counselling session to explore why he feels the way he does? Talking it out might be beneficial.

For *Negative Reactions to Bariatric Surgery-Induced Weight Loss* fuelled by partner loss of control, a few OSF members strongly suggested seeking support from domestic abuse services:

The man you described is an abusive narcissist! […] Although it may not be physical (yet), what you have described is emotional and mental abuse. You can find a safe place/haven by using the support systems available in every country for women just like you.

#### Re-evaluating relationships

Re-evaluating relationships, of both a platonic and romantic nature, was a frequently cited strategy to manage BS saboteurs. Making established boundaries clear, particularly in response to *Feeder Behaviours*, was commonly mentioned:

I believe it would be more beneficial for you to ask him to help you by eating or cooking away from you. This is a really sensible request.

OSF members who took on the aforementioned advice revealed finding it beneficial in mitigating BS saboteurs’ *Feeder Behaviours*:

My hubby and I have spoken in a calmer manner now. From here on out, there won’t be any more snacking in our bedroom.

Moreover, for *Feeder Behaviours* seemingly underpinned by unintentional and innocuous motivations, a few OSF members suggested that others re-evaluate the types of activities that were previously fundamental to the relationship and instead propose more BS-friendly activities:

If it [feeder behaviour] is coming from a place of love and ignorance, try inviting your friends to engage in non-food-related activities […] We socialise so much over food but that doesn’t have to be the case.

In contrast, for sabotage attempts deemed to be underpinned by more intentional and malicious motivations, several OSF members encouraged others to end no longer constructive platonic relationships:

It’s time to make a decision for your mental health and cut your friend out. You already made a decision for your physical health by getting bariatric surgery.

OSF members who adopted this strategy recounted finding it beneficial to their mental health:

After much deliberation, I decided to avoid contact with my friend for my own mental health. I know that I will require every last bit of it over the next six months. Although this sucks, this is for ME!

Similar recommendations were made regarding romantic relationships, with many OSF members encouraging others to assess whether their partnerships add value to their lives:

Do you want to instantly lose 150-200 pounds? Kick your partner to the curb! […] You might need to reconsider your relationship if you want to be happy in the long run.

#### Putting yourself first

Lastly, several OSF members reminded others of the importance of putting yourself first when faced with BS saboteurs:

Don’t allow this [partner’s sabotaging attempts] to undermine your goals. In the end, this [bariatric] surgery and weight loss are about you. Don’t allow others to make it about themselves, as your partner is doing.

Regarding *Feeder Behaviours*, while many OSF members would have preferred those in their support network to change their dietary behaviours akin to theirs, they believed that such an expectation is neither realistic nor fair. Instead, such OSF members stressed the importance of putting yourself first by taking control of your own BS destiny:

You have to do this [adhere to bariatric surgery guidelines] for yourself at the end of the day. Although it would be wonderful if everyone around us changed their diets and eating habits as a result of our [bariatric] surgery, this is neither feasible nor fair to them.

As a result of being sabotaged, OSF members described many coping strategies. These included shutting people out, retaliation, educating BS saboteurs, seeking alternative sources of support, re-evaluating relationships and putting themselves first.

### Transcending theme: The Online Support Forum as a Source of Substituted Social Support and Place of Solace

The OSF members therefore shared their experiences of being sabotaged through *Feeder Behaviours*, *Negative Reactions to Bariatric Surgery-Induced Weight Loss* and learning *Strategies to Avoid and/or Manage Bariatric Surgery Saboteurs*. As a result of this sharing process, many OSF members perceived *The Online Support Forum as a Source of Substituted Social Support and Place of Solace*:

In the past few days, this online support forum has helped me more than anyone else in real life ever has. I’m so happy to have found this online support forum and become part of this community.

A lot of OSF members sought substituted social support in lieu of the limited support they received from their support networks in real life:

Hi all, I’m back for some support because I can actually feel mine fading more quickly than I’m losing weight.

In response to these substituted social support requests, other OSF members offered positive reinforcements, sympathy and validation, as well as resonated with others:

I 100% relate to what you’ve just described. My ex-husband was exactly like that.

Furthermore, in response to learning that others have also encountered BS saboteurs, several OSF members reported feeling less alone:

I don’t feel as alone anymore because I know that other people have experienced similar problems as me.

## Discussion

This study explored OSF members’ experience of BS saboteurs post-BS. Three main themes were identified from the data: *Feeder Behaviours*, *Negative Reactions to Bariatric Surgery-Induced Weight Loss* and *Strategies to Avoid and/or Manage Bariatric Surgery Saboteurs*. Transcending these was the theme *The Online Support Forum as a Source of Substituted Social Support and Place of Solace*.

The first theme described OSF members’ experience of being sabotaged via *Feeder Behaviours*. In the context of weight management, a ‘*feeder*’ is defined as someone who offers others food even when they are not hungry (Ogden et al., 2020). Like Quirke-McFarlane and Ogden’s (2024) qualitative study, OSF members suggested that *Feeder Behaviours* were either explicit or implicit. Moreover, although their samples did not fully consist of individuals who had undergone BS, participants in Romo and Dailey’s (2014) and Romo’s (2018) qualitative studies similarly recounted how their support network’s feeder behaviours made weight management efforts more challenging.

Most OSF members articulated perceived feeder behaviour motivations. The unintentional and intentional feeder behaviour motivations support that of the wider literature (Andrews, 1997; Broderick, 1993; Ogden et al., 2020; Quirke-McFarlane and Ogden, 2024). For example, BS patients in Quirke-McFarlane and Ogden’s (2024) qualitative study similarly perceived affection and guilt avoidance as unintentional feeder behaviour motivations and envy, insecurity, jealousy, own weight management and spite as intentional feeder behaviour motivations.

An additional form of sabotage described in the second theme was *Negative Reactions to Bariatric Surgery-Induced Weight Loss*. A few OSF members mentioned being made aware explicitly or implicitly by family and friends that they preferred them at their pre-BS weight, a finding corroborating Griauzde et al. (2018). Given such negative reactions, it is perhaps unsurprising that some OSF members revealed that family and friends told them to stop losing weight. Although their samples did not include individuals who had undergone BS, Hindle and Carpenter (2011), Thomas et al. (2008a) and Thomas et al. (2008b) similarly reported how after substantial weight loss, family and friends of people living with obesity would comment that they looked sick or too thin and try to encourage them to stop dieting and lose weight.

OSF members reported several consequences of *Negative Reactions to Bariatric Surgery-Induced Weight Loss*. BS patients in Azizi et al.’s (2020) qualitative study comparably reported that their partner’s negative reactions to changes in their physical appearance made adherence to recommended dietary restrictions more challenging. Moreover, in line with previous research, many OSF members reported experiencing changes in their friendships, such as ostracism, due to their BS-induced weight loss (Bocchieri et al., 2002; Ficaro, 2018; Romo, 2018). Further, regarding romantic relationships, it was commonly acknowledged by OSF members that BS and all the associated changes can cause such relationships to breakdown, with many citing the potential for separation or divorce. Such observations support that of recent research (Bramming et al., 2021; Bruze et al., 2018). In their study of two large Swedish cohorts with long-term follow-up, Bruze et al. (2018) discovered that BS was associated with an increased incidence of divorce/separation.

Moreover, OSF members provided a variety of perceived motivations underpinning *Negative Reactions to Bariatric Surgery-Induced Weight Loss*. The perceived motivations behind *Negative Reactions to Bariatric Surgery-Induced Weight Loss* that OSF members classified as malicious corroborate that of the wider literature (Andrews, 1997; Crocker et al., 1998; Marques and Paez, 1994; Quirke-McFarlane and Ogden, 2024). For instance, Crocker et al. (1998) posited that people may sabotage others whose behaviours they perceive as threatening, such as weight loss, in an attempt to maintain social network members’ self-esteem and power inequalities, as well as to reassert group values.

The third theme described a multitude of *Strategies to Avoid and/or Manage Bariatric Surgery Saboteurs*. These included shutting people out, retaliation, educating BS saboteurs, seeking alternative sources of support, re-evaluating relationships and putting yourself first, all of which find reflection in theories of coping (Finset et al., 2002; Folkman and Lazarus, 1988). Whilst their sample did not consist of individuals who had undergone BS, Faw’s (2014) qualitative study similarly reported how young adults attempting to lose weight used strategies such as deception, ignoring and confrontation to manage non-support. Additionally, in terms of re-evaluating relationships, Faw (2014) also found that participants chose to create social plans that would limit temptations in an attempt to mitigate potential non-support. Regarding the coping strategy of educating BS saboteurs, participants in Romo’s (2018) qualitative study comparably reported providing family and friends with reassurance. Moreover, participants in Liebl et al.’s (2016) qualitative study attributed ending no longer constructive relationships/marriages, friendships and/or family relations and putting themselves first to their BS success.

Many OSF members perceived *The Online Support Forum as a Source of Substituted Social Support and Place of Solace*. This transcending theme highlighted the inadequacy of support from OSF members’ families, friends and acquaintances, leading them to seek solace and support elsewhere. The notion of *The Online Support Forum as a Source of Substituted Social Support and Place of Solace* corroborates that of previous BS OSF research (Athanasiadis et al., 2021; Atwood et al., 2018; Das and Faxvaag, 2014; Koball et al., 2017; Robinson et al., 2020; Willmer and Salzmann-Erikson, 2018). Some OSF members in Athanasiadis et al.’s (2021) qualitative study disclosed how being a member of the OSF made them feel understood and like they ‘*belong somewhere*’ (p. 4599). In this study, akin to others, social support came in many alternative forms, including the provision of positive reinforcements (Atwood et al., 2018; Koball et al., 2017), sympathy (Atwood et al., 2018) and validation (Atwood et al., 2018). Furthermore, in this study, learning about other OSF members’ encounters and struggles with BS saboteurs made many others in a similar situation feel less alone. Though not concerning BS saboteurs, OSF members in Athanasiadis et al.’s (2021) study comparably cited ‘*I am not alone as many are going through the same struggles*’ (p. 4599).

## Conclusion

This study explored OSF members’ experience of BS saboteurs post-BS. However, it is not without limitations. As thread replies from only one publicly available, active BS OSF available in the English language were analysed, the extent to which the results are generalisable to additional BS OSFs is unclear. Future research should therefore explore thread replies from a broader range of OSFs to determine if findings are generalisable across different platforms and contexts. Additionally, although members were required to create an account to use the OSF, it is unclear whether the OSF verified that these members were real individuals and not bots. This lack of verification could compromise the authenticity and reliability of the data analysed. Thus, future research should investigate the impact of member verification processes on the authenticity and reliability of OSF data.

The findings from this study hold important implications. Firstly, they provide further insight into the under-researched phenomenon of BS saboteurs. More specifically, this study illustrated what behaviours BS patients constitute as sabotage, the consequences of sabotage, potential explanations for sabotage, as well as strategies to help prevent and/or mitigate sabotage. Together, these findings can be used to support BS patients and healthcare professionals in addressing and overcoming BS saboteurs and ultimately achieving desired and optimal BS outcomes. Secondly, the findings from this study highlight the importance of *The Online Support Forum as a Source of Substituted Social Support and Place of Solace*. To ensure OSFs remain open and safe, forum moderators should establish clear guidelines, actively monitor discussions to prevent harassment and bullying, provide support resources and regularly engage with members to seek feedback and foster a supportive community. Lastly, the findings from this study provide empirical evidence for Ogden and Quirke-McFarlane’s (2023) new model of negative social support in the context of weight management and its emphasis on sabotage, feeder behaviour and collusion.

## Supplemental Material

sj-docx-1-hpq-10.1177_13591053241305946 – Supplemental material for ‘Is anyone else’s husband trying to undermine them all the time?’: A reflexive thematic analysis of online support forum discussions about bariatric surgery saboteurs Supplemental material, sj-docx-1-hpq-10.1177_13591053241305946 for ‘Is anyone else’s husband trying to undermine them all the time?’: A reflexive thematic analysis of online support forum discussions about bariatric surgery saboteurs by Sophia Quirke-McFarlane and Jane Ogden in Journal of Health Psychology

sj-docx-2-hpq-10.1177_13591053241305946 – Supplemental material for ‘Is anyone else’s husband trying to undermine them all the time?’: A reflexive thematic analysis of online support forum discussions about bariatric surgery saboteurs Supplemental material, sj-docx-2-hpq-10.1177_13591053241305946 for ‘Is anyone else’s husband trying to undermine them all the time?’: A reflexive thematic analysis of online support forum discussions about bariatric surgery saboteurs by Sophia Quirke-McFarlane and Jane Ogden in Journal of Health Psychology
