# Supplementary material for: ‘Is anyone else’s husband trying to undermine them all the time?’: A reflexive thematic analysis of online support forum discussions about bariatric surgery saboteurs
Source: J Health Psychol. 2024 Dec 29;30(14):4349–65. doi: 10.1177/13591053241305946 (PMC12678657; doi:10.1177/13591053241305946)
Supplement: sj-docx-1-hpq-10.1177_13591053241305946 – Supplemental material for ‘Is anyone else’s husband trying to undermine them all the time?’: A reflexive thematic analysis of online support forum discussions about bariatric surgery saboteurs [file sj-docx-1-hpq-10.1177_13591053241305946.docx]

**Supplementary Table 1.** Search Terms Utilised to Identify Bariatric Surgery Online Support Forums.

| Internet search engine | Search terms |
| --- | --- |
| Google | Bariatric surgery online support group OR Bariatric surgery Internet support group OR Bariatric surgery online support forum OR Bariatric surgery internet support forum OR Bariatric surgery forum OR Bariatric surgery message board OR Bariatric surgery discussion group |
|  | Weight loss surgery online support group OR Weight loss surgery internet support group OR Weight loss surgery online support forum OR Weight loss surgery internet support forum OR Weight loss surgery forum OR Weight loss surgery message board OR Weight loss surgery discussion group |
| Bing | Bariatric surgery online support group OR Bariatric surgery Internet support group OR Bariatric surgery online support forum OR Bariatric surgery internet support forum OR Bariatric surgery forum OR Bariatric surgery message board OR Bariatric surgery discussion group |
|  | Weight loss surgery online support group OR Weight loss surgery internet support group OR Weight loss surgery online support forum OR Weight loss surgery internet support forum OR Weight loss surgery forum OR Weight loss surgery message board OR Weight loss surgery discussion group |
