# Supplementary material for: ‘Is anyone else’s husband trying to undermine them all the time?’: A reflexive thematic analysis of online support forum discussions about bariatric surgery saboteurs
Source: J Health Psychol. 2024 Dec 29;30(14):4349–65. doi: 10.1177/13591053241305946 (PMC12678657; doi:10.1177/13591053241305946)
Supplement: sj-docx-2-hpq-10.1177_13591053241305946 – Supplemental material for ‘Is anyone else’s husband trying to undermine them all the time?’: A reflexive thematic analysis of online support forum discussions about bariatric surgery saboteurs [file sj-docx-2-hpq-10.1177_13591053241305946.docx]

**Supplementary Table 2**

*Generated Thread Search Terms*

| Search terms | | | | |
| --- | --- | --- | --- | --- |
| Antagonism | Discourage | Hurtful | Misunderstand | Sabotage |
| Callous | Disparaging | Impair | Mock | Saboteur |
| Collude | Disrupt | Insecure | Nag | Scrutinise |
| Compromise | Envious | Insensitive | Nagging | Scrutinize |
| Conflict | Envy | Jealous | Negative | Scrutiny |
| Conspire | Exhortation | Jealousy | Obstruct | Threatened |
| Control | Feeder | Judgemental | Paranoia | Thwart |
| Criticise | Friction | Malicious | Paranoid | Undermine |
| Criticize | Hinder | Manipulate | Problematic | Unhelpful |
| Disagreement | Hostile | Microaggressions | Resistant | Unsupportive |

*Note*. Thread search terms were generated from prior participant interviews, the existing literature, and input from a bariatric surgery patient advocate.
